# Familial partial lipodystrophy type 2 and obesity, two adipose tissue pathologies with different inflammatory profiles

**DOI:** 10.1186/s13098-023-01055-4

**Published:** 2023-04-21

**Authors:** Guillaume Treiber, Marie-Paule Gonthier, Alice Guilleux, Samir Medjane, Oriane Bonfanti, Muriel Cogne, Olivier Meilhac, Estelle Nobecourt

**Affiliations:** 1 Department of Endocrinology, Diabetes and Nutrition, GHSR, Centre Hospitalo-Universitaire de la Réunion, Saint-Pierre, La Réunion, France; 2 Université de La Réunion, INSERM, UMR 1188 Diabète Athérothrombose Thérapies Réunion Océan Indien (DéTROI), Plateforme CYROI, Saint-Denis de La Réunion, France; 3 Centre d’Investigation Clinique – Epidémiologie Clinique (CIC-EC) U1410 INSERM, Centre Hospitalo- Universitaire de la Réunion, La Réunion, France; 4 Délégation à la Recherche Clinique et à l’Innovation de La Réunion (DRCI), Centre Hospitalo- Universitaire de la Réunion, Saint-Pierre, La Réunion, France

**Keywords:** Inflammation, Dunnigan, Adipose tissue, Insulin resistance, Adipocytokines

## Abstract

**Introduction:**

The transition to metabolically unhealthy obesity (MUO) is driven by the limited expandability of adipose tissue (AT). Familial Partial Lipodystrophy type 2 (FPLD2) is an alternative model for AT dysfunction that is suitable for comparison with obesity. While MUO is associated with low-grade systemic inflammation, studies of inflammation in FPLD2 have yielded inconsistent results. Consequently, comparison of inflammation markers between FPLD2 and obesity is of great interest to better understand the pathophysiological defects of FPLD2.

**Objective:**

To compare the levels of inflammatory biomarkers between a population of patients with FPLD2 due to the same ‘Reunionese’ *LMNA* variant and a population of patients with obesity (OB group).

**Methods:**

Adiponectin, leptin, IL-6, TNF-α and MCP-1 plasma levels were measured by enzyme-linked immuno assays for 60 subjects with FPLD2 and for 60 subjects with obesity. The populations were closely matched for age, sex, and diabetic status.

**Results:**

Metabolic outcomes were similar between the two populations. Adiponectinemia and leptinemia were lower in the FPLD2 group than in the OB group (p < 0.01 for both), while MCP-1 levels were higher in the FPLD2 than in the OB group (p < 0.01). Levels of other inflammatory markers were not significantly different.

**Conclusions:**

Insulin-resistant patients with FPLD2 and obesity share common complications related to AT dysfunction. Inflammatory biomarker analyses demonstrated that MCP-1 levels and adiponectin levels differ between patients with FPLD2 and patients with obesity. These two AT pathologies thus appear to have different inflammatory profiles.

**Supplementary Information:**

The online version contains supplementary material available at 10.1186/s13098-023-01055-4.

## Introduction

Systemic low-grade inflammation in obesity is implicated as a primary marker of adipose tissue (AT) dysfunction (1), which then leads to adverse cardio-metabolic complications (2). In metabolically unhealthy obesity (MUO), cytokine profile changes include an increase in pro-inflammatory markers like IL-6, TNF-α, MCP-1 and high-sensitivity C-reactive protein (hs-CRP) with a concomitant decrease in anti-inflammatory markers like adiponectin (2–3). IL-6, TNF-α, MCP-1 and adiponectin are secreted by adipocytes and can be used to measure inflammation in AT (1–4), and the limited expandability of this AT is a key feature of the transition from metabolically healthy obesity to MUO. Familial Partial Lipodystrophy type 2 (FPLD2) [MIM#151,660] is a suitable alternative model to better understand the consequences of the limited expandability of AT (5). FPLD2 is a rare genetic disease caused by the ubiquitous expression of pathogenic *LMNA* variants in all cells (6,7). Previous conflicting studies have suggested that FPLD2 is associated with a pro-inflammatory state (8–11). In patients bearing the ‘typical’ *LMNA* p.(Arg482Trp/Gln) variants, hs-CRP and TNF-α plasma levels were significantly elevated compared levels BMI- and sex-matched controls (8,10). However, for other inflammatory biomarkers such as resistin, IL-6 and IL-1β, systemic levels did not differ between groups (8–9). These conflicting results might be explained, at least in part, by the small sample size of this rare disease group, or by the high heterogeneity of the control group. Interestingly, as described for obesity, adiponectinemia was lower in FPLD2 patients compared to healthy subjects (8,9,12). Both FPLD2 and obesity shared the same complications related to insulin resistance and inflammation, including diabetes, dyslipidaemia, cardiovascular disease and non-alcoholic fatty liver disease (NAFLD) (13,14). Therefore, a comparison of inflammatory biomarkers between subjects with obesity and FPLD2 is of great interest.

The aim of the present study was to measure and compare the levels of key AT-related inflammatory biomarkers in subjects with obesity to levels in FPLD2 patients having the same pathogenic *LMNA* ‘Reunionese’ variant.

## Materials and methods

### Patients

We described in Reunion Island (France), the largest cohort worldwide of subjects with FPLD2 caused by a unique *LMNA* pathogenic variant p.(Thr655Asnfs*49) (NM_170707.4:c.1961dup) called ‘Reunionese’ variant (15). This ‘Reunionese’ variant consists of a G insertion at codon 654 in prelamin-A-specific exon 11 (NM_170707.4:c.1961dup) (16). Consequently, the prevalence of FPLD2 in Reunion is particularly high (around 1/8,500 inhabitants) (15). We measured inflammatory biomarkers in the serum of 60 patients diagnosed with FPLD2 and genotyped for the ‘Reunionese’ variant (FPLD2 group). Six patients were homozygous, and 54 patients were heterozygous for the variant. We measured the same biomarkers in the serum of 60 patients with a body mass index (BMI) corresponding to a overweight (BMI > 25 kg/m^2^) or obese (BMI > 30 kg/m^2^) diagnosis (OB group). The groups were matched for age, sex, and diabetic status as closely as possible. The exclusion criteria were: age under 18 years, a familial partial lipodystrophy not attributed to the “Reunionese” variant. All patients provided written informed consents for molecular analyses. The study was performed according to local and European Union ethical rules (N°ID-RCB: 2021-A00105-36) and has been approved by the Institutional Ethical Committee of Sud Mediterranee IV, France.

### Data collection

Patient screening and follow-up were performed at the Reunion Island Lipodystrophy Competence Centre, part of the French National Reference Network for Rare Diseases of Insulin Secretion and Insulin Sensitivity. The attending physician conducted patient interviews and clinical examinations to collect medical history, demographic data, and clinical data. The clinical data collected were age, gender, total body weight, BMI and percentage body fat mass (%BFM) and treatment history for diabetes and dyslipidaemia. The biological data collected included HbA1c, insulin resistance determined by the homeostasis model assessment index (HOMA-IR), hs-CRP, total cholesterol (TC), triglycerides (TG), high density lipoprotein-cholesterol (HDL-C), low density lipoprotein-cholesterol (LDL-C) and kidney parameters: glomerular filtration rate was calculated using the CKD-EPI equation and urine albumin/creatinine ratio was measured on a urine sample. The presence of NAFLD was assessed by liver ultrasound. Levels of the liver enzymes aspartate aminotransferase (AST) and alanine aminotransferase (ALT) were measured. Liver fibrosis was assessed non-invasively. FIB-4 scores were calculated with the following formula: [age (years) × AST (IU/L)]/[platelet count (109/L) × ALT (IU/L)]. APRI score s were calculated with the following formula: [AST (IU/L)/platelet count (109/L)] × 100). The liver stiffness measurement (LSM) was performed using transient elastometry (Fibroscan 430, Echosens SAS, Paris, France). High-risk fibrosis was defined by FIB4 > 3.25, and/or APRI > 1.5 and/or F3-F4 fibrosis stage as assessed by the LSM. Hypertension was defined by blood pressure ≥ 140/90 mmHg or the use of antihypertensive drugs. Cardiac / vascular atherosclerotic diseases were defined by the presence of ischemic coronary disease or stroke, carotid stenosis or lower limb atherosclerosis as determined by Doppler ultrasound.

### Blood collection

Samples of serum were collected in 2 mL tubes without additive, aliquoted and stored at -80 °C prior to analysis. Levels of adiponectin, leptin, IL-6, TNF-α and MCP-1 were measured by ELISA. Assays were performed on 100 µL samples in 96-well plate for IL-6, MCP-1 (Human IL-6 and MCP-1/CCL2 Uncoated ELISA Kits Invitrogen ThermoFischer Scientific USA) and TNF-α (human TNF-α uncoated ELISA Kit Ebioscience USA) or diluted with purified water for leptin (dilution 1:20) (human leptin coated ELISA Kit Ray Biotech USA) and adiponectin (dilution 1:2500) (human adiponectin Coated ELISA Kit TECO Medical Group Switzerland). The sensitivity of the assays was 2.0 pg/mL for leptin and IL-6, 2.3 pg/mL for TNF-α, 7.0 pg/mL for MCP-1 and < 0.6 ng/mL for adiponectin. Absorbance was measured at 450 nm with a FLUOstar Omega Microplate Reader (BMG LABTECH GmbH Germany).

### Statistical analysis

Qualitative variables were described using frequency and percentages. Quantitative variables were described using medians and interquartile ranges. Bivariate comparisons of qualitative variables were assessed with the Chi-Square or the Fisher’s exact test, as appropriate. Quantitative variables were compared with the Mann and Whitney test. Due to the multiplicity of tests, the False Discovery Rate method correction was applied. P values were two-tailed and considered significant when lesser than 0.05. Pearson correlations were measured between the different metabolic and anthropometric parameters: a linear correlation was strong if the correlation coefficient |r|> 0.7; medium if 0.5 <|r|< 0.7 ; and low if 0.1 <|r|< 0.5 =. Statistical analyses were performed using R software version 4.0.3.

## Results

### FPLD2 and OB subjects displayed similar metabolic complications

The FPLD2 and OB groups were similar in age and sex composition (Table 1). As expected, weight, BMI and %BFM were lower in subjects with FPLD2 than in OB subjects (all p < 0.01). The two groups had a similar median HOMA-IR index, above 2.5, indicating an insulin-resistant state. The two groups did not differ in pre-diabetes or diabetes prevalence, age at onset of diabetes, or glycemic control (HbA1c). However, TG levels were significantly higher (p ≤ 0.01) and HDL-C levels significantly lower (p = 0.02) in the FPLD2 group compared to the OB group, despite similar use of lipid-lowering therapy. The prevalences of NAFLD, hypertension, cardiovascular diseases, and micro/macro-albuminuria were statistically similar between the groups.

**Table 1:** Clinical parameters and complications in patients from FPLD2 group harbouring the p.(Thr655Asnfs*49) pathogenic variant in comparison with OB population

	p.(Thr655Asnfs*49)	OB group	
	FPLD2 group		P-value*
**Age (years)**	39.5 [29.0-49.5]	40 [31.0-50.3]	0.78
**Sex (W/M)**	50/10 83.3%/16.7%	49/11 81.7%/18.3%	1
**Anthropometrics parameters**			
Weight (kg)	63.7 [55.5–77.7]	95.2 [79.9-107.9]	< 0.01
BMI (kg/m2)	25.1 [22.2–28.7]	35.0 [28.9–42.1]	< 0.01
Body fat mass (kg)	16.6 [11.7–23.9]	39.9 [28.0-51.9]	< 0.01
Waist circumference (cm)	86.0 [75.0–94.0]	106 [92.0-120.0]	0.006
Waist/Hip ratio	0.97 [0.93-1.00]	0.95 [0.92–0.98]	0.4
**Metabolic parameters**			
**Diabetes**	26 (43.3%)	24 (40.0%)	0.88
**Prediabetes**	20 (58.8%)	13 (37.1%)	0.28
HOMA-IR	3.9 [2.4–7.5]	4.2 [3.2–5.8]	0.78
**Dyslipidemia (a)**	50 (83.3%)	42 (71.2%)	0.36
Triglycerides (g/L)	1.4 [1.1-2.0]	1.1 [0.9–1.5]	< 0.01
LDL-cholesterol (g/L)	1.1 [0.8–1.3]	1.1 [0.8–1.4]	0.57
HDL-cholesterol (g/L)	0.4 [0.3–0.5]	0.5 [0.4–0.5]	0.02
Lipid-lowering therapy (a)	14 (24.1%)	10 (16.7%)	0.57
**Organ dysfunction**			
**Hepatic status**			
NAFLD (US)	47 (82.4%)	45 (78.9%)	0.87
FIB-4	0.7 [0.4–0.9]	0.6 [0.5–0.8]	0.85
High risk of hepatic fibrosis (b)	3 (5.2%)	4 (7.4%)	0.78
**Cardiovascular disease**			
Hypertension	19 (32.2%)	16 (26.7%)	0.78
Stroke	3 (5.0%)	1 (1.6%)	0.75
Ischemic coronary disease	4 (6.6%)	4 (6.6%)	1
Carotid stenosis > 50%	3 (5.0%)	1 (1.6%)	0.75
Lower limb atherosclerosis	1 (1.6%)	0 (0%)	0.79
Cardiovascular atherosclerotic disease (c)	14 (23.7%)	10 (16.7%)	0.64
**Kidney disease**			
Micro-/macro-albuminuria	15 (26.8%)	12 (23.5%)	0.92
Clearance (ml/min/1,73)	96 [82-111.5]	93 [80-103.3]	0.57

If not stated otherwise data are number (%) or median (Interquartile range). Prediabetes was defined by impaired fasting glucose (fasting plasma glucose between 6.1 and 6.9 mmol/L) and/or impaired glucose tolerance (plasma glucose 2 h-OGTT between 7.8 and 11.0 mmol/L).(a) Use of lipid-lowering agents (statin, ezetimib or fibrates), (b) High-risk liver fibrosis was defined by the presence of NAFLD on US with FIB-4 > 3.25 and/or APRI > 1.5 and/or F3-F4 fibrosis stage assessed by the liver stiffness measurement, (c) Cardiovascular atherosclerotic disease was defined by either a previous diagnosis of ischemic coronary disease or stroke, carotid stenosis > 50% or lower limb atherosclerosis assessed by Doppler US and/or a Coronary Artery Calcium (CAC) score > 11 in patients under 50 years of age or > 101 in patients over 50 years of age. *P-values were adjusted using False Discovery Rate method. (BMI) Body mass index, (HOMA-IR) homeostasis model assessment of insulin resistance, (NAFLD) nonalcoholic fatty liver disease, (M) Men and (W) Women, (US) liver ultrasound.

The FPLD2 and OB groups with diabetes were similar in age and sex composition (Table 2). As expected, weight and BMI were lower in subjects with FPLD2 and diabetes than in OB subjects (all p < 0.01). Median age at onset of diabetes and glycemic control (HbA1c) did not differ between the two groups. There was no difference in regard of the type of anti-diabetic treatment used between the two populations. Nine FPLD2 subjects and 4 subjects in OB group were under insulin therapy. Finally, there was no significant difference in micro-angiopathic (micro-/macro-albuminuria, peripheral neuropathy and retinopathy) and macro-angiopathic / cardiovascular complications.

**Table 2:** Clinical parameters and complications in patients from FPLD2 group harbouring the p.(Thr655Asnfs*49) pathogenic variant with diabetes in comparison with OB population with diabetes

	p.(Thr655Asnfs*49) FPLD2 group with diabetes	OB group with diabetes	
	n = 26	n = 24	P value*
**Age (years)**	46 [37.3–55.0]	49 [39.8–55.0]	0.95
**Sex (W/M)**	23/3 (88.5%) / (11.5%)	19/5 (79.2%) / (20.8%)	0.67
**Anthropometrics parameters**			
Weight (kg)	64.2 [56.4–79.5]	81.7 [76.3–93.0]	< 0.01
BMI (kg/m2)	25.3 [23.7–29.3]	29.6 [28.0-34.3]	< 0.01
Body fat mass (kg)	18.4 [14.8–28.4]	32.9 [25.6–39.0]	< 0.01
**Diabetes parameters**			
Age at onset of diabetes (years)	38.5 [26.3–43.0]	38.5 [35.8–48.5]	0.55
Metformin	20 (76.9%)	14 (70.0%)	0.91
Sulfonylureas	5 (19.2%)	7 (41.1%)	0.41
DPP-4i	4 (15.4%)	2 (12.5%)	0.94
GLP-1 RA	3 (11.5%)	4 (23.5%)	0.66
Insulin therapy	9 (34.6%)	4 (23.5%)	0.71
HbA1c (%)	7.5 [6.9–8.5]	7.3 [6.9-8.0]	0.66
**Dyslipidemia**			
Triglycerides (g/L)	1.4 [0.9–1.6]	1.6 [1.3–2.9]	0.11
LDL-cholesterol (g/L)	0.9 [0.6–1.1]	1.0 [0.8–1.4]	0.32
HDL-cholesterol (g/L)	0.4 [0.3–0.4]	0.4 [0.3–0.5]	0.2
Lipid-lowering therapy (a)	13(50.0%)	7 (29.2%)	0.46
**Hepatic status**			
NAFLD (US)	24 (96.0%)	18 (78.3%)	0.41
High risk of hepatic fibrosis (b)	2 (8.3%)	1 (4.8%)	1
**Cardiovascular disease**			
Hypertension	12 (46.2%)	10 (41.7%)	1
Cardiovascular atherosclerotic disease (c)	10 (40.0%)	6 (25.0%)	0.66
**Kidney disease**			
Micro-/macro-albuminuria	9 (36.0%)	7 (35.0%)	1
Clearance (ml/min/1,73)	93.0 [80.0-112.0]	94.0 [81.0-110.0]	1
**Peripheral neuropathy (d)**	7 (28.0%)	2 (8.3%)	0.56
**Retinopathy**	6 (23.1%)	2 (8.3%)	0.64

If not stated otherwise data are number (%) or median (Interquartile range). Prediabetes was defined by impaired fasting glucose (fasting plasma glucose between 6.1 and 6.9 mmol/L) and/or impaired glucose tolerance (plasma glucose 2 h-OGTT between 7.8 and 11.0 mmol/L).(a) Use of lipid-lowering agents (statin, ezetimib or fibrates), (b) High-risk liver fibrosis was defined by the presence of NAFLD on US with FIB-4 > 3.25 and/or APRI > 1.5 and/or F3-F4 fibrosis stage assessed by the liver stiffness measurement (c), Cardiovascular atherosclerotic disease was defined by either a previous diagnosis of ischemic coronary disease or stroke, carotid stenosis > 50% or lower limb atherosclerosis assessed by Doppler US and/or a Coronary Artery Calcium (CAC) score > 11 in patients under 50 years of age or > 101 in patients over 50 years of age (d) Peripheral neuropathy was assessed by the Semmes–Weinstein mono-filament test or DN4 score. *P-values were adjusted using False Discovery Rate method. (BMI) Body mass index, (DDP-4I) Dipeptidyl peptidase-4 inhibitors, (GLP-1 RA) Glucagon-like peptide-1 receptor agonist, (HOMA-IR) homeostasis model assessment of insulin resistance, (NAFLD) nonalcoholic fatty liver disease, (M) Men and (W) Women, (US) liver ultrasound.

### Inflammatory markers profiles differ between subjects with FPLD2 and obesity

**Fig. 1:**
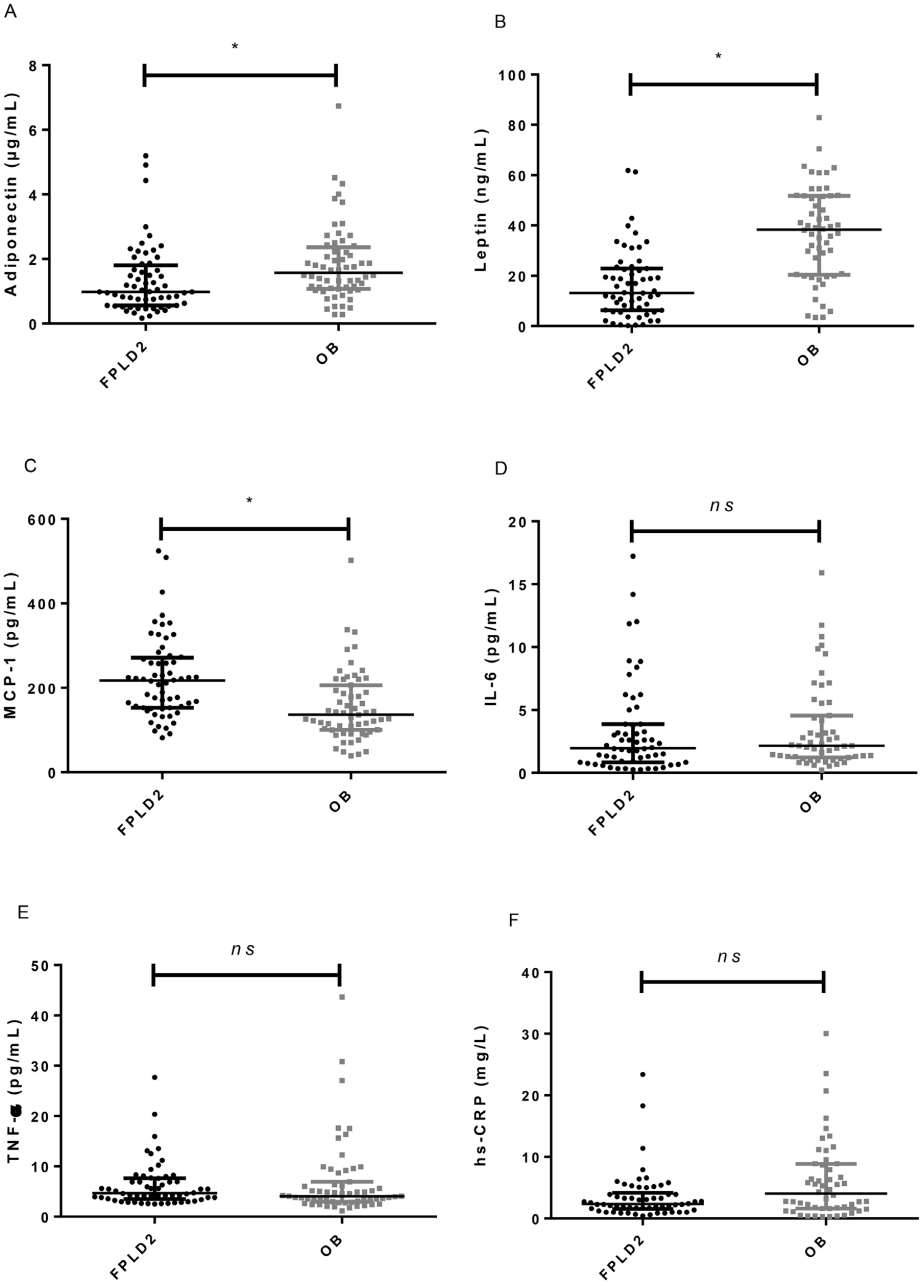
Adiponectin (A), leptin (B), MCP-1 (C), IL-6 (D), TNF-α (E) and hs-CRP (F) serum levels comparison between FPLD2 (n = 60) and OB (n = 60) subjects. Data are median (Interquartile range). * p < 0.01, P-values were adjusted using False Discovery Rate method

**Fig. 2:**
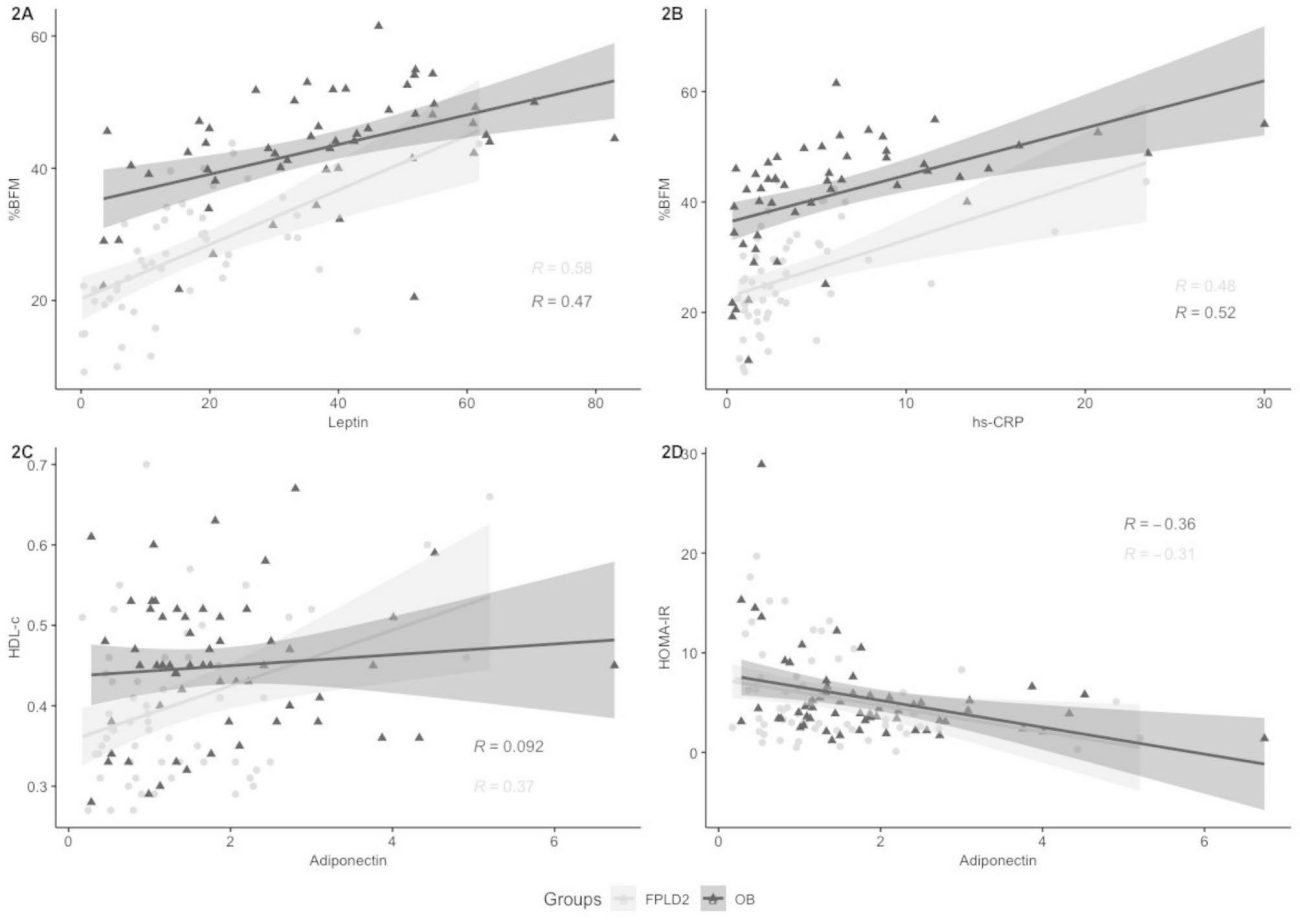
Significant Pearson correlations between the different metabolic and anthropometric parameters in patients with FPLD2 due to the “Reunionese” *LMNA* variant (n = 60) and in patients with obesity (n = 60). (HOMA-IR) homeostasis model assessment of insulin resistance, (%BFM) % of body fat mass was assessed by Bioelectrical Impedance Analysis

Adiponectinemia and leptinemia were lower in FPLD2 patients than in OB subjects (p < 0.01 for both) (Fig. 1A and B), while MCP-1 levels were significantly higher in the FPLD2 group (p < 0.01). No differences were observed in levels of hs-CRP, IL-6 and TNF-α between the groups. As expected, leptin levels showed a significant positive correlation with BMI and %BFM in FPLD2 (respectively R = 0.71, p < 0.001 and R = 0.58, p < 0.001) and OB subjects (R = 0.45, p < 0.001 and R = 0.47, p < 0.001, respectively) (Fig. 2A). We also found that hs-CRP levels correlated positively with BMI and %BFM in FPLD2 (R = 0.48, p < 0.001 and R = 0.48, p < 0.001, respectively) and OB subjects (R = 0.55, p < 0.001 and R = 0.52, p < 0.001, respectively) (Fig. 2B). Adiponectin, MCP-1, IL-6 and TNF-α levels were not correlated with BMI and %BFM in either group. In the FPLD2 group, adiponectinemia was positively correlated with HDL-C level (R = 0.37, p = 0.003) and negatively associated with the HOMA-IR (R = -0.31, p = 0.018) (Fig. 2C and D). In OB group, HOMA-IR was negatively associated with adiponectinemia (R = -0.36, p = 0.004) and positively correlated with hs-CRP level (R = 0.29, p = 0.037). Data did not indicate any other significant correlation between HDL-C level or HOMA-IR and other inflammatory biomarkers (Additional File 1).

## Discussion

In this study, we measured inflammatory biomarkers between two distinct insulin resistant populations related to AT dysfunction: one with FPLD2 and the other with obesity. Results show that subjects with FPLD2 had significantly lower leptin and adiponectin levels and higher MCP-1 levels in comparison with subjects with obesity, despite similar metabolic complications. The prevalence of diabetes, used as a matching criterion, could not be analysed. Data also show no statistical difference in the levels of other cytokines including IL-6, TNF-α and hs-CRP between both groups.

Adiponectin is an interesting surrogate marker of insulin sensitivity. Adiponectin is also an anti-inflammatory adipocytokine due to its ability to attenuate pro-inflammatory signalling pathways (17). In our study, as reported previously, both FPLD2 and OB groups had extremely low levels of adiponectin (8,9,12). However, hypoadiponectinemia was significantly more pronounced in FPLD2 potentially as a consequence of adipocytes apoptosis and oxidative stress induced by the accumulation of mutated prelamin in FPLD2 (7) in addition to insulin resistance. Hypoadiponectinemia has been associated with endothelial tissue dysfunction, which is unsurprising given that adiponectin provides a protective barrier against monocyte adhesion and thus protection against subsequent endothelial cells inflammation (18). Thus, in patients with lipodystrophy, hypoadiponectinemia could aggravate endothelial cells dysfunction induced by the accumulation of mutated pre-lamin in these cells and increased oxidative stress (11). Vascular abnormalities associated with a poor metabolic environment may be responsible for the extremely high risk of cardio-vascular diseases in this population. Interestingly, in lipoatrophic mice model, the glycaemic status was improved with recombinant adiponectin (19). This finding suggests that adiponectin could be a promising treatment for FPLD2 in improving metabolic and vascular functions. Interestingly, MCP-1 levels were particularly highly elevated in our FPLD2 group. Adiponectin has been reported to have a reducing effect on MCP-1 production in adipocytes (20) and endothelial cells (21). MCP-1 is produced by a variety of cell types (macrophages, endothelial cells, adipocytes) and is incriminated in vascular dysfunction through the recruitment of immune cells and the release of pro-inflammatory cytokines. In obesity, MCP-1 production increases in proportion to visceral adiposity expansion (22). In lipodystrophy, the overexpression of p.R482W prelamin-A in transfected human coronary artery endothelial cells was shown to increase MCP-1 expression significantly (11). In lipodystrophy, the overexpression of p.R482W prelamin-A in transfected human coronary artery endothelial cells was shown to increase MCP-1 expression significantly (11). We found no evidence of a relationship between MCP-1 and adiponectin levels in the Dunnigan population (data not shown). Nonetheless, considering the interactions already identified between these two biomarkers, it may be prudent to investigate in appropriate cellular and animal models whether MCP-1 overexpression in endothelial and adipose tissues in FPLD2 may be related to hypoadiponectinemia in FPLD2.

In a previous study, we analyzed glucose homeostasis in a sample of 102 patients with the ‘Reunionese’ FPLD2 variant and compared measures with those from 22 unaffected adult relatives (23) with similar BMI values. Median leptin levels in unaffected subjects did not differ from leptin levels in the ‘Reunionese’ FPLD2 subjects (23). The leptin levels observed in this previous study (23) were similar to those observed in the present FPLD2 group. Furthermore, our results also show that leptinemia in Dunnigan subjects was significantly lower than in OB subjects, underscoring the strong relationship between leptin production and body fat mass. In patients with FPLD2, recombinant leptin treatment clearly improves weight management and insulin sensitivity while reducing free fatty acid (FFA) levels (24) which is consistent with the impact of hypoleptinemia on these metabolic abnormalities.

Our work is the first to measure inflammatory biomarkers in the largest cohort worldwide of FPLD2 patients with the pathogenic ‘Reunionese’ variant of *LMNA*. We found that systemic levels of IL-6, hs-CRP, and TNF-α in FPLD2 subjects were comparable to those in obese, insulin-resistant patients. For these two insulin-resistant AT diseases, no human study has compared inflammatory biomarkers. These findings could imply that FPLD2 is a pro-inflammatory illness, but such an inference should be viewed with caution. The switch to MUO in hypertrophic AT is characterized by deterioration of the immuno-modulatory system, especially in visceral AT, and an imbalanced profile with an excess of pro-inflammatory immune cells (25). Many inflammatory biomarkers are secreted by adipocytes and immune cells in the AT environment, including hs-CRP, IL-1β, IL-6, and TNF-α. There are undoubtedly more markers that contribute to the development of MUO (1–4,26). In contrast, the development of MUO is associated with a reduction in the expression of anti-inflammatory and/or insulin-sensitizer cytokines (adiponectin, IL-10) (26,27).

Inflammation in obesity is strongly linked to insulin resistance; and inflammatory markers interfere with insulin signaling pathways through activation of JNK and MAPK or IKK/NF-κB pathways (28,29). As with obesity, lipodystrophy appears to be associated with changes that include a decline in immuno-modulating T cells, and a shift in the phenotype of macrophages toward a pro-inflammatory profile (30). Levels of TNF-α (8,10), IL-6 (10,11), hs-CRP (9,10), and IL-1β (10) were significantly higher in the relatively smaller FPLD2 subjects than in healthy subjects. Herrero et al. (31) observed elevated systemic inflammation in lipoatrophic aP2-nSREBP-1c transgenic mice, with levels increased 1.9-fold for TNF-α, 10.2-fold for IL-6 and 1.4-fold for MCP-1 as compared to wild-type littermates. In contrast, the anti-inflammatory cytokine IL-10 was 3.3-fold lower in lipodystrophic mice. This inflammation resulted from generalized macrophage infiltration into all AT depots, with substantial numbers of T and B lymphocytes (31). Additional basic research data and clinical data are needed to expand on the few studies that do indeed suggest a pro-inflammatory state in lipodystrophy (8–11). To enable comparison of inflammation to be made with FPLD2 subjects in the future, we are establishing a bio-collection of blood samples from lean, non-mutated control subjects that are matched for sex, age, and BMI. Finally, we plan to assess levels of biomarkers in blood samples to provide an overview of the systemic inflammation in FPLD2.

In whole-body histological examination of lipoatrophic aP2-nSREBP-1c transgenic mice, systemic inflammation was identified by exclusively AT pro-inflammatory cytokine expression with a substantial number of infiltrating macrophages in AT (31). Interestingly, no inflammation was described in other tissues, including the severely steatotic livers. However, Bidault et al. (11) demonstrated that overexpression of p.R482W prelamin-A in transfected human coronary artery endothelial cells was associated with an increase in MCP-1, IL-6, and IL-8 expression in this tissue. Unfortunately, the design of our study did not include direct measures of endothelial dysfunction. The hypothesis that systemic inflammation in FPLD2 originates from sources other than AT inflammation is being debated. Further research in suitable preclinical models is required to determine the degree to which ubiquitous mutated prelamin expression is responsible for inflammation in specific tissues (AT, endothelium). Such work could help to elucidate the genesis of cardio-metabolic outcomes in this population.

The differences in fat mass and body composition between individuals with overweight or obesity could possibly interfere with the degree of inflammation in OB group, and we performed a comparison of the inflammatory biomarker levels between these two subgroups. (Additional file 2). As expected, in regard with previous results, we found higher levels of leptin and hs-CRP in subjects with obesity than subjects with overweight. Furthermore, there was no significant difference in levels for the other biomarkers between the two groups.

FPLD2 and obesity are two insulin resistant diseases in relation with the AT dysfunction. In our study, TG level is significantly higher and HDL-C level is significantly lower in FPLD2 group in comparison with OB group, despite similar HOMA-IR index. Genetic AT dysfunction with severe inappropriately buffers lipid flux and elevated serum FFA and TG, may explain these differences in lipid profile between FPLD2 and OB group. Lastly, TG / HDL-C level parameters influence the level of insulin resistance/sensitivity, they are not the only ones (32). This could explain the difference in TG and HDL-C levels between the two populations in our results.

In conclusion, our study confirms that insulin-resistant patients with FPLD2 and obesity share common complications related to AT dysfunction. Inflammatory biomarker analyses demonstrated that MCP-1 levels and adiponectin levels differ between patients with FPLD2 and patients with obesity. These two AT pathologies thus appear to have different inflammatory profiles. This difference may be related to the impact of the *LMNA* mutation on both adipose tissue and endothelial cells in FPLD2. Particularities of systemic inflammation in FPLD2 could be linked to the origin of the cytokines and may have an impact on the development of disease complications. These last points remain to be explored.

## Electronic supplementary material

Below is the link to the electronic supplementary material.

Supplementary Material 1

Supplementary Material 2

## Data Availability

The datasets used and/or analyzed during the current study are available from the corresponding author on reasonable request.

## Abbreviations

AT: adipose tissue
BMI: body mass index
%BFM: percentage body fat mass
FFA: Free Fatty Acid
FPLD2: Familial Partial Lipodystrophy type 2
HDL-C: high density lipoprotein-cholesterol
HbA1c: hemoglobin A1c
HOMA-IR: insulin resistance determined by the homeostasis model assessment index
hs-CRP: high-sensitivity C-reactive protein
LDL-C: low density lipoprotein-cholesterol
MUO: metabolically unhealthy obesity
NAFLD: non-alcoholic fatty liver disease
TC: total cholesterol
TG: triglycerides
VAT: visceral adipose tissue.
