# Supplementary material for: Familial partial lipodystrophy type 2 and obesity, two adipose tissue pathologies with different inflammatory profiles
Source: Diabetol Metab Syndr. 2023 Apr 21;15:77. doi: 10.1186/s13098-023-01055-4 (PMC10120265; doi:10.1186/s13098-023-01055-4)
Supplement: Supplementary file 1 — Supplementary Material 1 [file 13098_2023_1055_MOESM1_ESM.docx]

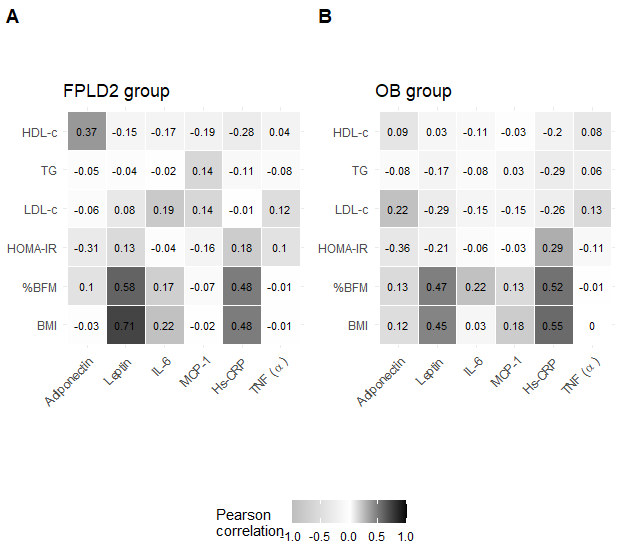


**Additional file 1**: Heatmap of Pearson correlation between the different metabolic and anthropometric parameters in patients with FPLD2 due to the “Reunionese” *LMNA* variant (n = 60) (A) and in patients with obesity (n = 60) (B). (BMI) Body mass index, (HOMA-IR) homeostasis model assessment of insulin resistance, (%BFM) % of body fat mass was assessed by Bioelectrical Impedance Analysis.
