# Supplementary material for: Familial partial lipodystrophy type 2 and obesity, two adipose tissue pathologies with different inflammatory profiles
Source: Diabetol Metab Syndr. 2023 Apr 21;15:77. doi: 10.1186/s13098-023-01055-4 (PMC10120265; doi:10.1186/s13098-023-01055-4)
Supplement: Supplementary file 2 — Supplementary Material 2 [file 13098_2023_1055_MOESM2_ESM.docx]

|  | **OB group subjects with 25>BMI>30kg/m2 n= 21** | **OB group subjects with BMI>30kg/m2 n= 39** | *p value* |
| --- | --- | --- | --- |
| **Age (years)** | 46 [38-55] | 36 [29.5-48.5] | 0.01 |
| **Sex (W/M)** | 12/9 | 37/2 | 0.001 |
| **Anthropometrics parameters** |  |  |  |
| Weight (kg) | 77 [70.3-77.7] | 104 [94.4-117.7] | 0.001 |
| BMI (kg/m2) | 28 [26.1-29.0] | 39 [35.2-43.4] | 0.003 |
| **Inflammatory markers** |  |  |  |
| Adiponectin (g/L) | 1.4 [1.1-1.8] | 1.8 [1.1-2.5] | 0.31 |
| IL-6 (pg/mL) | 2.2 [1.3-5.6] | 2.2 [1.3-5.6] | 0.96 |
| MCP-1 (pg/mL) | 142.9 [115.0-193.3] | 126.2 [96.6-201.9] | 0.65 |
| TNF-a (pg/mL) | 3.9 [3.0-6.2] | 4.2 [3.1-7.8] | 0.96 |
| Leptin (µg/L) | 20.8 [10.5-38.1] | 42.9 [32.1-54.6] | 0.001 |
| hs-CRP (mg/L) | 1.5 [0.5-2.8] | 6.3 [2.8-11.0] | 0.001 |

Supplemental Table 1: Clinical parameters and inflammatory biomarkers levels comparison in patients in OB group with overweight (25<BMI<30kg/m2) in comparison with patients in OB population with obesity (BMI>30kg/m2). If not stated otherwise data are number (%) or median (Interquartile range).
